# Energy Consumption, Colour, Texture, Antioxidants, Odours, and Taste Qualities of Litchi Fruit Dried by Intermittent Ohmic Heating

**DOI:** 10.3390/foods9040425

**Published:** 2020-04-03

**Authors:** Xiaohuang Cao, Md. Nahidul Islam, Wanxiu Xu, Jianping Chen, Bimal Chitrakar, Xuejing Jia, Xiaofei Liu, Saiyi Zhong

**Affiliations:** 1 College of Food Science and Technology, Guangdong Ocean University, Zhanjiang 524088, China; caoxhfood@163.com (X.C.); cjp516555989@126.com (J.C.); jiaxj@gdou.edu.cn (X.J.); liuxf@gdou.edu.cn (X.L.); 2 Department of Food Science, Aarhus University, Agro Food Park 48, DK-8200 Aarhus N, Denmark; nahidul.islam@food.au.dk; 3 School of Engineering, Zhejiang Normal University, Jinhua 321004, China; bcxwx@126.com; 4 College of Food and Technology, Jiangnan University, Wuxi 214122, China; bimalchitrakar@gmail.com

**Keywords:** e-tongue, e-nose, ohmic heating, phenols, litchi fruits

## Abstract

To reduce the cost of dried litchi fruit, the processing characteristics and physicochemical properties of litchi were investigated using drying by intermittent ohmic heating (IOH) (intermittent air drying (IAD)) generated by BaTiO_3_ resistance. Litchi fruit pulp were dried at 70 °C with an air velocity of 1.8 m/s; the drying intermittent profiles were as follows: (1) 20 min drying-on and 5 min drying-off; (2) 20 min drying-on and 10 min drying-off; and (3) 20 min drying-on and 15 min drying-off, which correspond to pulse ratios (PRs) of 1.2, 1.5, and 1.8, respectively. After drying, the water content, energy consumption, vitamin C content, total phenolic content, colour, taste, and odour qualities were assessed. The results suggested that IOH drying requires lower energy consumption and yields higher quality products. The energy consumption of intermittent air drying ranged from 341 kJ∙g^−1^ to 427 kJ∙g^−1^. The IAD of 1.2 and 1.5 PR reduced the browning of litchi fruits and gained better product quality. The major components of odour and tastes were explored in dried litchi. The rising PR of IAD enabled a lower retention of methane and sulphur-organic aroma and a higher assessing value of bitterness taste. This study revealed that BaTiO_3_ is suitable for IOH drying and it resulted in more merits of dried litchi fruit.

## 1. Introduction

Litchi fruit (*Litchi Chinensis Sonn.*) is produced largely in South China and contains high amounts of vitamin C, polyphenols, and sugar [1]. Its sweetness is favoured by consumers in China, and the polyphenols found in litchi helps to prevent incidence of heart disease and protect physiological metabolism [2]. However, processors encounter difficulties in the storage and transportation of litchi for the concentrated maturation of litchi fruit [3]. Processors extend shelf life and facilitate transportation through dehydration, which includes sun drying, freeze drying, air drying, and microwave drying [4,5,6]. Sun drying feathers uncontrollability and vulnerability from natural conditions [7]. Freeze drying, on the other hand, contributes to enrich product quality, while it requires high cost [8]; whereas microwave drying obtained lower cost but results in non-uniform products in terms of appearance, colour, and moisture content [9]. Vacuum drying requires a vacuum system, which increases the ultimate cost [10]. To obtain a high-quality product with low energy consumption, novel drying technologies are always getting priorities for drying industry.

Litchi fruits are mostly processed by air drying with a simple technology of conventional ohmic heating of a chromel-filament [6]. However, conventional ohmic heating (air drying) is shown to have low energy efficiency, take longer, and produce low product quality [11]. Additionally, the continuous ohmic resistant heat of air drying can degrade the antioxidant compounds, colour, flavour, and sensory qualities of litchi fruits [12]. Therefore, it is of great significance to obtain dried product with better colour, odour, nutrition, and low energy consumption in the drying of litchi fruit. However, Song C.F. et al. [6] assessed the sensory qualities of dried litchi fruits and found that the acceptance of dried product is not affected by various drying methods. Intermittent air drying (IAD) is known as an outstanding technique in food drying [12,13,14,15,16]. Intermittent air drying confirmed that IAD decreases the operation cost and increases yields of high-quality fruits and vegetables [13,14,15]. IAD allowed the easy movement of moisture from the interior to the surface of material spontaneously by temperature gradient [15]. Moreover, a temperature drop during the tempering period is found to reduce the nutrient degradation [16]. It is reported that intermittent dehydration decreased energy consumption in onion by 12% and obtained 17% energy savings in apple drying [17,18]. The ohmic heating of BaTiO3 resistance provides more merits of safety, clarity, and moderation with its resistance of positive temperature coefficient [18,19,20,21,22]. Using IOH, the IAD of litchi fruits is expected further to raise the retention of vitamin C and phenolics and to lower energy consumption. Far fewer reports can be found regarding IOH and related to IAD, and even less reports have attempted to assess the flavours and odours of dried litchi [23,24,25,26,27,28,29,30,31,32,33]. Therefore, an attempt has been taken to improve the colours, tastes, and odours and decrease the loss of vitamin C and phenolics during drying as well as lower energy consumption.

In this study, new knowledge was provided about IOH affecting the antioxidant substances, energy consumption, colours, tastes, odours, and water dynamics of litchi fruits.

## 2. Material and Methods

### 2.1. Materials

Fresh litchi fruit (20 kg) was received from Guangzhou Origin Food Science & Technology Company Ltd. within 24 h of delivery by using an ice-box. According to Chinese consumers’ preferences, litchi fruit (1 kg) was peeled and pitted along the fruit axis, and then the fruit pulp was stored at 4 °C in a refrigerator [23].

The heater element was equipped with the BaTiO_3_ resistance of a positive temperature coefficient that generates ohmic heating. BaTiO_3_ resistance (2000 W) was purchased from Tiancheng Thermistors Co., Ltd. In Daojiao town, Dongguan, Guangdong, China. BaTiO_3_ resistivity rises sharply with temperature in each given region, which gives constant temperature. The temperature of ohmic heating was determined by air speed, applied voltage, and resistance value.

### 2.2. Procedure

**Figure 1:**
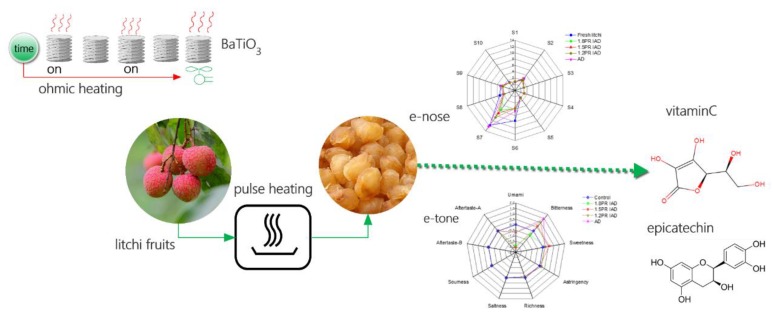
Flow sketch of litchi dried by drying schemes of different pulse ratios.

Litchi pulp (500 g, 4 mm thickness) was spread on perforated trays (40 × 30 cm), and then the trays were put into the air dryer for drying at 70 °C. A proportional integral differential (PID) program was used to control drying-on and drying-off and to measure the weight of dried samples online. All operations were depicted as flow in Figure 1, which consisted of three parts: material treatment, drying schemes, and processing evaluation.

Drying parameters were set at 70 °C with 1.8 m/s air velocity; the drying intermittent profiles were set for three variations, viz., 20 min drying-on and 5 min drying-off; 20 min drying-on and 10 min drying-off; 20 min drying-on and 15 min drying-off. In each drying scheme, when the sampling time (elapsed 50 min) arrived, the weight of each sample was recorded until drying ended viz. the dried mass of litchi weighed constant for 20 s. Meanwhile, dried samples (2–5 g) were randomly picked for moisture determination and quality assessing. The initial wet base moisture content was 86% (wet basis). All tests had been tested at Guangdong Ocean University (Zhanjiang, China) and all evaluations and measurements were repeated three times.

Pulse ratio (PR) values are important parameters affecting the drying kinetics and energy consumption. PR was expressed using following Equation (1) as shown by Cao, Chen, Islam, Xu, and Zhong [23]:

(1) $$\mathrm{PR}=\frac{t_{\mathrm{on}}+t_{\mathrm{off}}}{t_{\mathrm{on}}}$$

where PR is the pulse ratio of drying, t_on_ is the “on” time, and “t_off_” is the “off” time of air drying (min).

### 2.3. Proximate Composition Analyses

Dried samples (2–5 g) were picked for moisture determination using a hot air oven at 105 °C until constant weight, as described in the modified methods [24,25]. Protein was determined by the Kjeldahl method [23]. Total sugars were determined as in a previously described method [6].

### 2.4. Energy Consumption

The energy consumption in each part of the dryer was measured by the electrical energy meter (PF9800, Gigital Power Meter, Everfine Company, Hangzhou, China). The total energy consumed (electric energy for ohm heating, divided by sample) was calculated without consideration of ohmic resistance as shown by Li et al. [26].

### 2.5. Colour Evaluation

The surface colour was measured using a colorimeter with a measuring area 5 mm in diameter. Three parameters were obtained for the average of five measurements. The ideal L*, a*, and b* values of fresh pieces were considered as references in the following array; L* represents lightness, b* represents yellowness and blueness, and a* represents redness and greenness. Before measurement, calibration was done three times using a standard whiteboard. The colour of the fresh samples were measured to get three ideal parameters of *L*_0_, *a*_0_, and *b*_0_ values, and the colour difference was described as the colour change according to Islam et al. [27].

### 2.6. Determination of Texture

An evaluation of samples texture was achieved using a TX-XT Express texture analyser (Stable MicroSystems, Vienna Court, Lammas Road, Godalming GU7 1YL, United Kingdom) as described by Cao, Islam, Zhong, Pan, Song, Shang, Nie, Xu, and Duan [24]. The test program was a penetration of compressibility. A cylindrical probe (2 mm diameter) penetrated through the dried sample pulp. Pre-test, test, and post-test rates were set at 1, 1, and 20 cm/min, whereas the penetrate distance was 5 mm. A standard weight used for calibration was 1.0 kg.

### 2.7. Determination of Density

The mass of a 2–5 g dried sample was weighed firstly for *m*_1_. The mass of a porous basket submerged in water was weighed for *m*_2_. After weighing the mass of the dried sample and submerged basket, the sample was put into the basket and submerged in water quickly, and then the weighed mass of the sample-uploading basket was submerged in water for *m*_3_ within 5 s. The Cao density (Cd) was calculated by Equation (2):

(2) $$\mathrm{Cd}=\frac{m_{1}}{\left(m_{1}+m_{2}-m_{3}\right)/\rho_{w}}$$

where Cd represents the Cao Xiaohuang density of the sample, g/cm^3^; *m*_1_, represents the mass of the sample, g unit; *m*_2_ represents the mass of the basket submerged in water, g unit; *m*_3_ represents the mass of the sample-uploading basket submerged in water, g unit; *ρ_w_* represents the water density, when 0–40 °C, $\rho_{w}$ is approximately equal to 1.0 g/cm^3^. The Cao density (Cd) is applicable to determine the density of dried fruits containing sugars and fatty starches because their density is higher than that of water.

### 2.8. Determination of Vitamin C

Ten g sample was ground in a grinder machine (2000 C, 550 W, Taiyang electromechanical company, Jinhua, ZHE, China) and moved to a 500 mL flask containing 50 mL of distilled water. Then, 20% sulfuric acid solution (10 mL) and 1% starch solution (1 mL) were supplemented. The sample was titrated with a dropping of 0.01 M of I_2_ solution until it obtained a blue colour, which did not fade for 15 s. The consumed volume of I_2_ solution was used for assessing the vitamin C content (mg/kg of vitamin C, d.b(dried basis)) [24]. The method was verified using standard vitamin C.

### 2.9. Total Phenolic Compounds

Dried litchi pulps (10 g) were smashed with a grinder and mixed with 20 mL 80% acetone. Then, 10 mL of hydrochloric acid (HCl, 5 M) was added for hydrolysis in 3 h using a magnetic stirrer at 70 °C. The hydrolysed sample was vacuum filtered, and the residue was flushed and filtered again using acetone. The extract (1 mL) was mixed with 4 mL of distilled water and 2 mL of Folin and Ciocalteu’s reagent. Then, 7% Na_2_CO_3_ solution (10 mL) was injected and incubated for 10 min. Thus, incubated solution was used to determine absorbance at 760 nm using a spectrophotometer (UV752A, Shanghai precision instrument technology Co. Ltd., 1195 Pingliang Road, Shanghai, China). Calibration curves were depicted with six points by using a standard substance of gallic acid of 1 mg/L, 2 mg/L, 4 mg/mL, 6 mg/L, 8 mg/L, and 10 mg/L. The total phenolic compounds were calculated as mg/kg of dry matter and expressed as gallic acid equivalent [24].

### 2.10. E-Tongue Analysis

An electronic tongue (TS-5000Z, INSENT, Kanagawa, Tokyo, Japan) possessed nine taste sensors including sourness, astringency, sweetness umami, bitterness, saltiness, richness, after-taste-A, and aftertaste-B, as shown by Woertz et al. [28]. The concentration of taste is converted into electrical signals, and the electrical signals were then converted into an evaluation value of staff. Electronic tongue measurement was as follows: samples (1 g) were pasted by the glass mortar (8 × 10 cm) for 2 min. This paste (1 g) was mingled with 100 mL of distilled water and stirred for 5 min (1000 rpm). The mixture was centrifuged at 5000 rpm with a Centrifuge (SIGMA 3-18K, Sartorius, Göttingen, Germany). After 5 min, the supernatant (taste substances) was poured into the test cup (diameter 30 mm, height 35 mm) for electronic tongue measurement. Each sample was assessed four times; the measurement cycle consisted of measuring the reference solution followed by the sample solution, a short (2–4 s) cleaning step, and measurement of the aftertaste. Assessing the value from the voltage change (mV) of the inner and outer membranes of taste was directly proportional to the concentration of the taste substances and an evaluation value of staff was recorded for assessing.

### 2.11. E-Nose Analysis

Gas analysis was carried out by an Electronic nose, model PEN3 (Airsense Analytics GmbH, Schwerin, AV, Germany) [29,30]. The electronic nose was characterised by 10 sensors including aromatic (S_1_, Benzene), broad range (S_2_, oxynitride), aromatic (S_3_, amines), hydrogen (S_4_), aromatics (S_5_, aliphatic), broad methane (S_6_), sulphur-organic (S_7_), broad alcohol (S_8_), sulphur-chlorinate (S_9_), and methane-aliphatic (S_10_). The odour signals are converted into electrical signals by sensor array, and the signal ratio were transmitted to a computer. Then, 0.5 g of dried sample was chipped within 2 mm and put into a 15-mL centrifugal tube at the bottom. After loading, the centrifugal tube was sealed by a sealing sticker, and then it was bathed at 40 °C for 5 min. After bathing, one needle with a carbon filter was inserted at a 5 cm depth of the centrifugal tube for balancing gas and one needle with a 0.22 µm filter was inserted with a 3 cm depth of the centrifugal tube for determination of the odour signal. E-nose operation consisted of three steps: (1) first, clearing the sensor; (2) second, waiting for the sample injection, (3) third, assaying the sample odour. Parameters: clearing time 60 s, sampling time 60 s, waiting time 5 s, cavity air fluid 400 mL/min, and injection air fluid 16 mL/min.

### 2.12. Data Analysis

Analysis of variance (ANOVA) and multiple comparisons using the T-test method were performed on the test data using SPSS software (SPSS 20.0, IBM, Chicago, IL, USA). The significance level was *p* < 0.05, and the confidence level was 95%. All diagrams were drawn with Origin 8.0 software (OriginLab Corporation, Roundhouse Plaza, Suite 303 Northampton, Massachusetts, USA. All measurements were carried out in triplicate.

## 3. Results and Discussion

### 3.1. Water Dynamics

**Figure 2:**
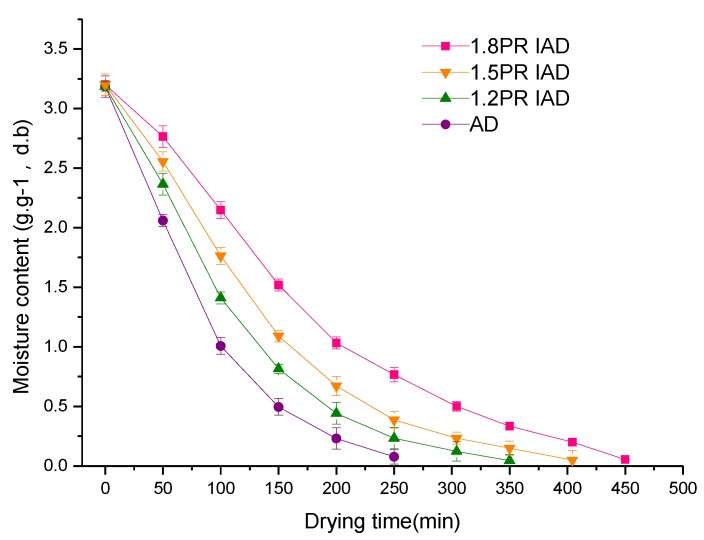
Water dynamics of litchi dried by drying schemes with different pulse ratios. Note: AD is air drying, IAD is intermittent air drying, PR is pulse ratio [(drying time + interval time)/drying time] in one cycle, average value ± standard deviation, d.b. is dried basis.

Figure 2 shows the drying curve characteristics of dried samples at different schemes of drying-on and drying-off. Dehydration curves are characterised by a difference of the dehydration ratio and time, resulting in variations of cost and quality. These dehydration kinetics are related to the temperature, material properties, and air velocity. Drying rate is controlled by the moisture diffusion from the inside to outside of the material. Air drying (AD) was found to possess a high drying rate and short cumulative time (drying-on time plus drying-off time). IAD yields a low drying rate and longer time (drying-on time plus drying-off time). This result demonstrated that IOH resulted in a long drying time, while continuous ohmic heating causes a high drying rate. The reason was low temperature and stopping air flow during the drying-off time, which decreased the moisture-escaping velocity, which was similar to that reported by earlier authors [31,32].

When AD started, the drying rate was constant at the beginning, which was found to be increased after some time and then decreased until it tends to zero. However, IAD showed a constant drying rate at the beginning, which then dropped until it reached zero. The drying time (450 min) at 1.8 PR was decreased by about 12% compared with that (400 min) at 1.5 PR. The fast-falling rate in the later period of 1.8 PR accounted for this result compared with the low-falling rate in 1.5 PR. From Figure 2, 1.2 PR of IAD enabled decreasing the time (350 min), while the drying time was 450 min for the AD of litchi fruits; the drying time was most reduced by 22%. These results demonstrated that IOH is better than continuous ohmic heating for the drying of litchi fruits. The mechanism behind it might be that the surface resistance to vapor was predominant in the early time period [33]; moisture diffusion inside litchi fruit that had the IAD drying rate mainly administered delivered more chances to promote water diffusion over the longer time period.

### 3.2. Drying Time and Energy Consumption

Table 1 lists the time and energy consumption of litchi fruits dried by different schemes using intermittent air drying. Although different IAD schemes required more cumulative time than AD in the processing of litchi fruits, IAD required more total drying-on time, with low energy consumption. In this sense, these data imply that IOH requires low cost for the drying of litchi fruits. Energy consumption of 1.5 or 1.8 PR was significantly lower than that for 1.2 PR in IAD. This means that increasing the interval (off-drying time) enabled mass transfer by a spontaneous process without energy provided by an oven. The drying of 1.2 PR decreased the cumulative drying-on time from 300 min to 250 min, which was reduced about by 17%. A large interval or high PR indicated a high drop of energy consumption, which was accompanied by a reduction in the molecular mobility. This result demonstrated that an increasing interval of IOH results in better energy consumption. The process of reducing the molecular mobility accounted for this phenomenon by temperature gradient to escape vapour from materials [31,32]. Table 1 presents the lowest energy consumption (427 kJ·g^−1^) of IAD and 488 kJ·g^−1^ energy consumption of AD. Energy consumption dropped by about 12%. This energy consumption saving is on account of some moisture transfer automatically to material surfaces and vacuum without energy supply from electric heating. Studies confirm that the drying temperature and pulse ratio impact the product quality and energy consumption [34]. The regulation of the pulse ratio is a smart strategy allowing high quality and low energy consumption in the ohmic heating drying of litchi fruits.

### 3.3. Colour Evaluation of Litchi Pulp

Table 2 lists the colour changes of litchi fruit dried by IAD at various pulse ratios. All values of lightness were different (*p* < 0.05) from those of fresh samples, which were shifted to red and yellow during air drying. This means that all thermal processing impaired the colour quality of products as earlier reported [23]. There was no significant difference between the 1.2 and 1.5 PR dried samples in lightness, whereas PR 1.8 drying resulted in L*, a*, and b* values of samples that were different from those of the AD-dried samples, and other PR IAD. This means that IOH increased lightness and reduced browning. It might be on the account of the lower temperature generated during interval that decreased the occurrence of charring and browning [35]. However, in IAD, browning (shifting to dark, red and yellow) grows in PR in IAD. Study confirmed this browning in AD and relieved charring in IAD [36]. The shift to red and yellow in IAD is obviously different (*p* < 0.05) from the continuous AD of litchi fruit. However, a further increasing PR of 1.8 enhanced the high values in lightness and redness values. In case of a yellow value, the b* value increased with the increase in PR, and a maximum b* value of 13.8 was found at continuous drying at 70 °C. This is ascribed to shortening the drying time by the exposure at high temperature. In other words, non-enzymatic browning was relieved for colour degradation through IOH.

The index *ΔE* value of the 1.2/1.5 PR treated sample was lower than that of the 1.2 PR IAD and AD sample of litchi fruits. After drying, the *ΔE* values had a significant difference (*p* < 0.05) across all the drying schemes. A high *ΔE* value represents a large colour change of dried samples between drying schemes. A maximum *ΔE* value of 15.11 demonstrated that the high PR of 1.8 impaired the colour quality more significantly (*p* < 0.05). From Table 2, IAD was found to relieve the brown colour, and browning decreased with the 1.2 and 1.5 pulse ratios. This result confirmed that IOH relieves browning. This is on account of non-enzymatic browning being formed easily by the high temperature and long drying time of litchi fruits.

### 3.4. Texture Evaluation of Litchi Pulp

**Figure 3:**
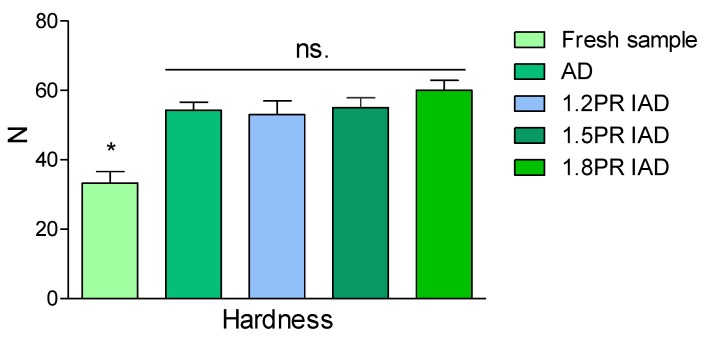
Hardness of samples dried by drying schemes with different pulse ratios. Note: AD is continuous air drying; 1.2 PR is an on 20 min and off 5 min pulse ratio of intermittent air drying, 1.5 PR is an on 20 min and off 10 min ratio of intermittent air dying, PR 1.8 is an on 20 min and off 15 min ratio; PR = [(on-drying time + off-drying time)/drying time] in one cycle, value of hardness is the average value ± standard deviation; asterisk (*) is significantly different (*p* < 0.05); n.s. is non-significant.

Hardness is an influencing parameter for the acceptability of samples. The hardness of litchi pulp was not statistically significant (*p* < 0.05), although there were slight changes in the hardness between four different dried samples (Figure 2). The main reason was that a mass of total sugar determined the texture and was less affected by ohmic heating. Figure 3 shows that the increase of hardness was significantly different from that of the fresh samples (*p* < 0.05). The reason for the results is that substances are concentrated by ohmic heating, which led to the increasing density of litchi [37,38]. The hardness of the dried samples is due to the glucose and pectin content [39] and the viscidity of litchi pulp. From the above discussion on intermittent air dying, IOH is an alternative of low cost and high quality in the processing of litchi pulp. Hot air allied with IOH in this research is a successful application to gain acceptable texture. Regarding the earlier reference, the hardness of litchi pulp increased with the rise of drying temperature [24]. However, this law concerning hardness is not sustained by our results in dried litchi fruits. The reason might be due to the litchi variety or different maturity. High temperature worsening the quality of dried litchi fruit has been shown in continuous ohmic heating. However, IOH allowed deceasing the drying time and ambient temperature, which accounted for this better result.

### 3.5. Vitamin C Evaluation of Litchi Pulp

**Figure 4:**
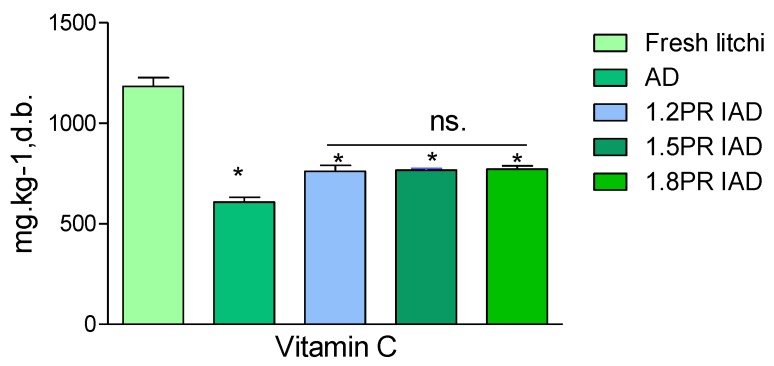
Vitamin C content of litchi dried by drying schemes with different pulse ratios. Note: AD is continuous air drying; the ratio 1.2 PR is an on 20 min and off 5 min pulse of intermittent air drying; the pulse ratio 1.5 PR is an on 20 min and off 10 min ratio of intermittent air drying; PR 1.8 is an on 20 min and off 15 min ratio; PR = [(on-drying time + off-drying time)/drying time] in one cycle; vitamin C value is the average value ± standard deviation; asterisk (*) is significantly different (*p* < 0.05); n.s. is non-significant; d.b. is dried basis.

Figure 4 shows the vitamin C content change of fresh litchi pulp and dried samples. There was a significant decrease of vitamin C (*p* < 0.05) after drying. The vitamin C content of samples was non-significantly (*p* < 0.05) different in 10 min-on and 15 min-off schemes. This implies that different schemes of ohmic heating drying allowed different levels of nutrition degradation. From Figure 4, about 50% of the vitamin C was retained under IAD. Continuous air drying and a long interval (off-drying time) caused severe degradation. These results ascertained that an appropriate PR of ohmic heating is suitable for better product quality. The reason might be that two factors, viz. processing temperature and the time of exposure to surrounding heat, caused vitamin C oxidation [40]. It is also claimed that oxidase enzymes remained active during drying, which catalyses vitamin C degradation, but the loss of vitamin C was most likely on account of non-enzymatic oxidation during drying [41]. A 30–50% retention in vitamin C in the microwave vacuum drying of litchi pulps was reported [42]. IAD preserves vitamin C by about 50%. When incorporating interval off-drying, the retention in vitamin C increased significantly by about 10% (*p* < 0.05), as compared with only continuous drying at 70 °C. It can be concluded that IOH as drying is superior to air drying litchi pulps and enables the promotion of vitamin C content.

### 3.6. Total Phenolic Evaluation of Litchi Pulp

**Figure 5:**
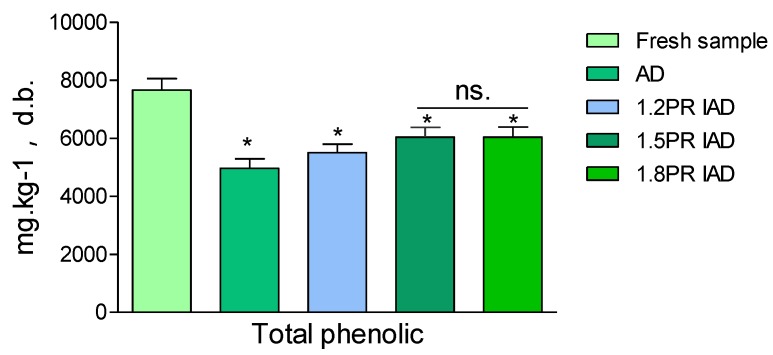
Total phenolic content of litchi dried by drying schemes with different pulse ratios. Note: AD is continuous air drying; ratio 1.2 PR is an on 20 min and off 5 min pulse of intermittent air drying, the pulse ratio 1.5 PR is an on 20 min and off 10 min of intermittent air dying, PR 1.8 is an on 20 min and off 15 min ratio; PR = [(on-drying time + off-drying time)/drying time] in one cycle, values of phenolic is average value ± standard deviation, asterisk (*) is significantly different (*p* < 0.05); n.s. is non-significant; d.b. is dried basis.

Figure 5 presents the content of phenolic compounds in litchi pulp dried by IAD, in comparison with fresh litchi fruits. Phenolic compounds are believed to be partially responsible for health protection [43]. After drying, the total phenolic content of dried samples decreased significantly (*p* < 0.05) from fresh samples [44]. Compared to only hot air drying, the total phenolic of the IAD-dried samples was statistically higher (*p* < 0.05) than that of the AD-dried sample. These results showed that interval ohmic heating increased the retention of total phenolic compounds. In other words, IAD enabled an average retention of 70–80% polyphenolic compounds, whereas only 60% of phenolic content was retained for continuous air drying. The possible reason might be the decreasing time of exposure to oxygen for phenolic decay in IAD [45]. There was no difference among the three intermittent air-drying schemes of litchi fruits. The reason might be interpreted as phenolic oxidation being retained by the sugar components.

### 3.7. E-Nose Profile of Litchi Pulp

**Figure 6:**
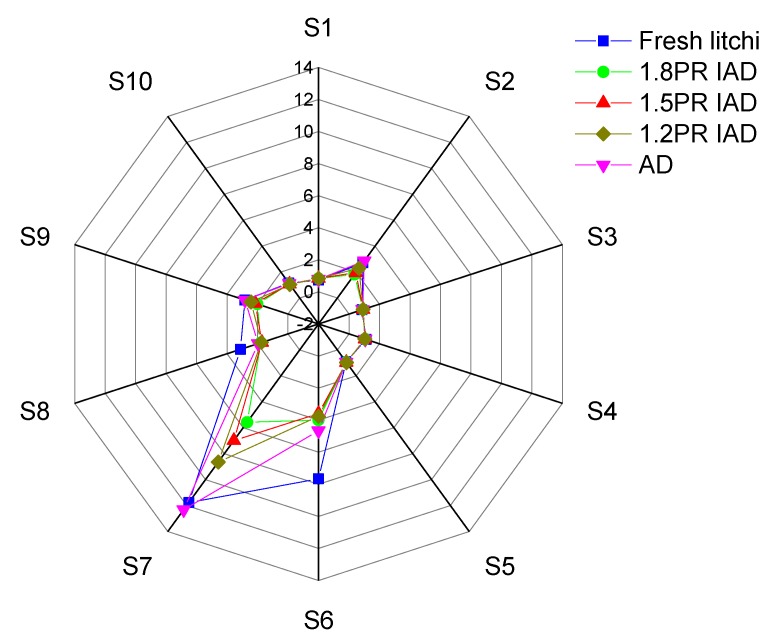
E-nose evaluation of litchi dried by drying schemes with different pulse ratios. Note: AD is continuous air drying; the ratio 1.2 PR is an on 20 min and off 5 min pulse of intermittent air drying, pulse ratio 1.5 PR is an on 20 min and off 10 min of intermittent air dying, PR 1.8 is an on 20 min and off 15 min ratio; PR = [(on-drying time + off-drying time)/drying time] in one cycle. S1 stands for benzene, S2 stands for oxynitride, S3 stands for amines, S4 stands for hydrogen, S5 stands for aliphatic, S6 stands for methane, S7 stands for sulphur-organic, S8 stands for broad-alcohol, S9 stands for sulphur-chlorinate, and S10 stands for methane-aliphatic.

Figure 6 showed the evolution of litchi odour dried by different drying schemes. After different intermittent air drying, S_7_ and S_6_ dominated dried litchi aroma, which referred to methane and sulphur-organic. The signals of these two odours significantly changed after drying, and these signals decreased with rise of the PR (pulse ratio) in air drying. This tendency and the characteristics of litchi fruit odours means that litchi contains more methane hydrate and sulphur-organic compounds. These results implied that the PR of ohmic heating is an important factor affecting odour. The methane odour might be from the dissociation of methane hydrate e.g., saccharose, fructose, and glucose. The sulphur-organic odour might be mercaptan, thioether, and thiophenol from sulphur replacing oxygen during drying [46]. The same results were found in vegetables and fruits which confirmed that sulphur-organic odour is the main odour components [47]. Dried litchi also maintained more methane odour. This might be related to the high content of sugars in litchi. From Figure 6, it can be seen that compared with methane, the sulphur-organic odour dropped quickly. This phenomenon originates from that sulphur-organic compounds (mercaptan, thioether, and thiophenol) are insoluble in water and have a low boiling temperature. These attributes were determined by the non-hydrogen bonds between molecules and water or between these molecules [48,49]. From Figure 6, the two main components of S_7_ and S_6_ decreased at the bottom of the signal profiles after 1.8 PR IAD and represent the maximum value in fresh litchi. This result suggested that the high PR value of ohmic heating decreased the retention of odours. In production, 1.2–1.5 PR might be appropriate for the conservation of litchi odour profiles.

### 3.8. E-Tongue Profile of Litchi Pulp

**Figure 7:**
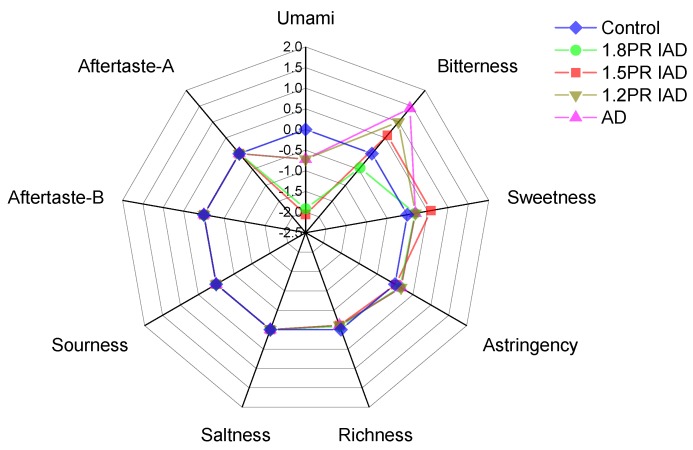
E-tongue evaluation of litchi dried by drying schemes with different pulse ratios. Note: AD is continuous air drying; the ratio 1.2 PR is an on 20 min and off 5 min pulse of intermittent air drying, the pulse ratio 1.5 PR is an on 20 min and off 10 min ratio of intermittent air dying, PR 1.8 is on 20 min and off 15 min; PR = [(on-drying time + off-drying time)/drying time] in one cycle.

Figure 7 shows the change of nine main tastes in litchi fruit dried with different schemes of ohmic heating. After drying, increasing bitterness values were discovered and decreasing sweetness values were found. This meant that the taste profile of litchi fruit was affected by different schemes in ohmic heating. This behaviour is ascribed to the oxidation and decomposition of litchi components, e.g., Maillard reaction and the decomposition of aldehydes and ketones [48]. Saltness, richness, astringency, sourness, aftertaste-B, and aftertaste-A were slightly affected by the air-drying profiles of ohmic heating. One possible reason is that the low content of salt in litchi fruits and glutamate regarding umami might be exhausted by the Maillard reaction [49]. The bitterness value increased with the decrease of pulse ratio (PR), and the maximum value occurred in the 1.8 PR IAD sample. This behaviour might be due to the long processing of ohmic heating increasing the heterocyclic compounds, which is related to bitterness. From Figure 7, three tastes (umami, bitterness, and sweets) were revealed to be affected by different air-drying schemes. Intermittent air drying showed that the appropriate drying of ohmic heating would be suitable to obtain nice tastes of litchi.

### 3.9. Based/Compositional Evaluation of Litchi Pulp

A high temperature of 70 °C led to browning and bitterness, which decreases the sensory scores. The main reason is that the Maillard reaction accelerates darkness and forms bitter substances. The maximum a*/b* values of the 1.8 PR dried sample are observed in Table 2. The reason might be that the longer time enhances burning using 1.8 PR during IAD. However, the IAD profiles show a relieving of browning compared to continuous air drying. These results implied that IOH is superior to continuous ohmic heating in the drying of litchi fruits. The reason might be that the interval of temper time softened the temperature, which the reduced browning of litchi.

Table 3 shows that the protein, total sugar, and moisture values were not different (*p* < 0.05) in dried samples; they corresponded to average values of 1.61, 92.76, and 16.86 (g/100g), respectively. Meanwhile, there was no difference (*p* < 0.05) in the density of the dried litchi fruits, which was equal to 1.32 g/cm^3^. After different IAD schemes, the water activity value of litchi was under 0.6, which means dried litchi possessed better stability. These results show that IAD is suited to the conservation of litchi fruits and implied that IOH has better continuous ohmic heating. As listed in Table 3, lower water activity values were observed—0.45 and 0.46 occurrence in 1.6 and 1.5 PR IAD—which were different (*p* < 0.05) from those in the IA samples. The reason might be that longer dehydration times cause less boundary water in litchi.

## 4. Conclusions

In this work, intermittent ohmic heating (IOH) with BaTiO_3_ resistance was carried out for drying litchi fruit, and we have profiled tastes, odours, vitamin C, phenolics, hardness, and energy consumption. New merits of IOH were discovered in relation to saving energy consumption, relieving browning, reducing umami, raising the bitterness, and decreasing methane and sulphur-organic odours. We found that long-time ohmic heating produced a bitter taste, and sulphur-organic odour is the main odour in processed litchi. BaTiO_3_ resistance with a positive temperature coefficient was found to be a suitable heating element in the drying of litchi.

The core mechanism of IOH is the automatic diffusion and evaporation during interval (off-drying time) without an external electrical energy supply, which saves energy. The thermodynamics of IOH is the main reason that it influences the vitamin C and phenolic content in litchi fruit. IOH in this study is an applicability in the drying industry, which allows further dehydration development.

## Figures and Tables

**Table 1:** Drying time and energy consumption of drying schemes of different pulse ratios.

Drying/ 70 °C	Cumulative Time/min	On-Drying Time/min	Off-Drying Time/min	Energy Consumption/(kJ∙g^−1^)
1.8 PR IAD	450	250	200	341 ± 0.42 ^a^
1.5 PR IAD	400	260	130	385 ± 0.51 ^b^
1.2 PR IAD	350	280	70	427 ± 0.11 ^c^
AD	300	300	0	488 ± 0.22 ^d^

Note: Different letters indicate significant differences (*p* < 0.05) in a column, average value ± standard deviation; AD is continuous air drying; ratio (1.2 PR) is an on 20 min and off 5 min pulse of intermittent air drying, the pulse ratio 1.5 PR is an on 20 min and off 10 min ratio of intermittent air dying, PR 1.8 is an on 20 min and off 15 min ratio; PR = [(on-drying time + off-drying time)/drying time] in one cycle.

**Table 2:** Colour change of litchi dried by drying schemes with different pulse ratios.

IAD/70 °C	PR	L *	a *	b *	*ΔE*
Fresh		62.73 ± 1.44 ^a^	−4.22 ± 0.17 ^d^	5.45 ± 0.37 ^e^	-
On 20 min/off 15 min	1.8	19.45 ± 1.35 ^c^	9.47 ± 0.13 ^a^	15.84 ± 0.28 ^a^	15.11 ± 0.44 ^a^
On 20 min/off 10 min	1.5	22.70 ± 1.13 ^b^	5.37 ± 0.25 ^c^	11.25 ± 0.52 ^c^	10.53 ± 0.52 ^c^
On 20 min/5 min	1.2	24.77 ± 1.24 ^b^	4.85 ± 0.25 ^c^	9.97 ± 0.35 ^d^	9.27 ± 1.55 ^c^
AD	-	18.60 ± 1.18 ^c^	7.86 ± 0.35 ^b^	13.80 ± 0.38 ^b^	11.29 ± 2.43 ^b^

Note: Different letters indicate significant differences (*p* ≤ 0.05) in a column, average value ± standard deviation; AD is continuous air drying; 1.2 PR is an on 20 min and off 5 min pulse ratio of intermittent air drying, 1.5 PR is an on 20 min and off 10 min of intermittent air dying, PR 1.8 is an on 20 min and off 15 min ratio; PR = [(on-drying time + off-drying time)/drying time] in one cycle.

**Table 3:** Approximate nutrition of litchi pulps dried by drying schemes with different pulse ratios.

Based Properties of Dried Litchi Fruits Using Intermittent Air Drying					
PR	Protein g/100g	Total Sugar g/100g, d.b.	Moisture Content g/100g, d.b.	Water Activity	Cao Density g/cm ^3^
1.8	1.70 ± 0.15 ^a^	92.15 ± 1.25 ^a^	16.25 ± 1.34 ^a^	0.45 ± 0.01 ^b^	1.32 ± 0.03 ^a^
1.5	1.75 ± 0.17 ^a^	93.45 ± 1.44 ^a^	16.22 ± 1.25 ^a^	0.46 ± 0.01 ^b^	1.31 ± 0.02 ^a^
1.2	1.45 ± 0.13 ^a^	93.41 ± 1.15 ^a^	17.34 ± 1.42 ^a^	0.47 ± 0.01 ^a^	1.33 ± 0.02 ^a^
AD	1.55 ± 0.11 ^a^	92.05 ± 1.22 ^a^	17.65 ± 1.25 ^a^	0.46 ± 0.01 ^a^	1.31 ± 0.01 ^a^
Average value	1.61 ± 0.15	92.76 ± 1.30	16.86 ± 1.44	0.46 ± 0.01^a^	1.32 ± 0.15

Note: Different letters in a column are significantly different (*p* < 0.05), average value ± standard deviation; AD is air drying, PR is the pulse ratio of intermittent air drying; AD is continuous air drying; ratio 1.2 PR is an on 20 min and off 5 min pulse of intermittent air drying, pulse ratio 1.5 PR is an on 20 min and off 10 min ratio of intermittent air dying, PR 1.8 is an on 20 min and off 15 min ratio, PR = [(on-drying time + off-drying time)/drying time] in one cycle; d.b. is dried base.
